# Does hyperthermia increase skeletal muscle damage from eccentric exercise?

**DOI:** 10.1186/2046-7648-4-S1-A149

**Published:** 2015-09-14

**Authors:** John W Castellani, Edward J Zambraski, Michael N Sawka, Maria L Urso

**Affiliations:** 1 United States Army Research Institute of Environmental Medicine, Natick, MA, USA

## Introduction

Rhabdomyolysis is often associated with novel physical exercise and exertional heat illness in warm temperatures. It is possible that accentuated hyperthermia might partially be responsible for these observations, but direct evidence is lacking. Previous research has not manipulated skeletal muscle temperature and evaluated its impact on muscle damage induced by a standardized eccentric exercise (ECCEX) challenge. It was hypothesized that local muscle heating (HEAT) applied just before ECCEX will cause greater skeletal muscle functional loss and damage compared to ECCEX performed without muscle heating (CON).

## Methods

Eight volunteers (age, 22.5 ± 4.1 yr; height, 169.5 ± 10.8 cm; body mass, 76.2 ± 12.6 kg), serving as their own control, completed two elbow flexor ECCEX trials; in one trial the biceps were heated > 40°C and in the other trial there was no heating (muscle temperature ~36°C). Each ECCEX trial consisted of two bouts of 24 maximal eccentric contractions of the elbow flexor muscles in one arm. HEAT was applied with shortwave diathermy (100 W) for 15 minutes immediately before the first ECCEX bout and for 2 minutes in between each bout. Individuals were followed for 10 days after each ECCEX session. There was a 6-week washout period and the volunteer then completed the 2^nd ^ECCEX trial in the other arm. Functional data (maximal voluntary isometric contraction (MVC), soreness, bicep circumference, range of motion (ROM) are presented as mean ± SD at the 48-h post-ECCEX period and blood indices of muscle damage and adaptation (creatine kinase (CK), myoglobin, heat shock proteins (HSP), interleukins) at 72-h.

## Results

The MVC decreased by 41 ± 17% and 46 ± 20% in the CON and HEAT trials, respectively, 48h post-exercise. Soreness (100-point visual scale) increased 41 ± 26 and 43 ± 29 points in the CON and HEAT trials, respectively. Bicep circumference increased by 4% in both trials, and the relaxed ROM in the elbow flexor decreased by 30% in the CON and HEAT trials. Serum CK peaked 72-h following ECCEX (CON: 6289 ± 10407; HEAT: 5486 ± 6229 IU/L, increase of 3,700%) as did serum myoglobin (CON: 362 ± 483; HEAT: 355 ± 373 ug/L, increase of 1,200%). No statistically significant differences were observed between CON and HEAT for any functional or muscle damage marker measurement. Furthermore, there were no differences between treatments for HSP 27 and 70, as well as interleukins 1b and 10.

## Conclusion

The results suggest that elevating skeletal muscle temperature before eccentric exercise does not alter functional, subjective, and blood indices of muscle damage. In addition, the elevated muscle temperature did not alter the HSP and interleukin response following eccentric contractions. These data suggest that acute heat exposure does not increase the risk of heat-related rhabdomyolysis.

